# Mendelian Inconsistent Signatures from 1314 Ancestrally Diverse Family Trios Distinguish Biological Variation from Sequencing Error

**DOI:** 10.1089/cmb.2018.0253

**Published:** 2019-05-08

**Authors:** Prachi Kothiyal, Wendy S.W. Wong, Dale L. Bodian, John E. Niederhuber

**Affiliations:** ^1^ Inova Translational Medicine Institute, Inova Health System, Falls Church, Virginia.; ^2^ Department of Public Health Sciences, School of Medicine, University of Virginia, Charlottesville, Virginia.

**Keywords:** de novo mutations, inherited deletions, long interspersed nuclear elements (LINE), Mendelian-inconsistent calls (MIC), population-specific deletions, quality control, repeats, short interspersed nuclear elements (SINE), trio sequencing, whole-genome sequencing.

## Abstract

**Next-generation sequencing enables advances in the clinical application of genomics by providing high-throughput detection of genomic variation. However, next-generation sequencing technologies, especially whole-genome sequencing (WGS), are often associated with a high false-positive rate. Trio-based WGS can contribute significantly towards improved quality control methods. Mendelian-inconsistent calls (MIC) in parent–child trios are commonly attributed to erroneous sequencing calls, as the true de novo mutation rate is extremely low compared with MIC incidence. Here, we analyzed WGS data from 1314 mother, father, and child trios across ethnically diverse populations with the goal of characterizing MIC. Genotype calls in a trio can be used to assign different signatures to MIC. MIC occur more frequently within repeats but show varying distribution and error mechanisms across repeat types. MIC are enriched within poly-A/T runs in short interspersed nuclear elements. Alignability scores, allele balance, and relative parental read depth vary among MIC signatures and these differences should be considered when designing filters for MIC reduction. MIC cluster in germline deletions and these MIC also segregate with population. Our results provide a basis for making decisions on how each MIC type should be evaluated before discarding them as errors or including them in alternative applications. With the reduction of sequencing cost, family trio whole genome and exome analysis are being performed more routinely in clinical practice. We provide a reference that can be used for annotating MIC with their frequencies in a larger population to aid in the filtering of candidate de novo mutations.**

## 1. Background

Whole-genome sequencing (WGS) is increasingly used for the high-throughput detection of genomic variation (Bentley et al., 2008) and is enabling advances in the clinical application of genomics. However, WGS is often associated with a nonnegligible rate of incorrectly identified sequence variants due to sequencing errors instead of true genomic variation. An error rate of ∼0.01–0.1 per base sequenced using an Illumina platform (Meacham et al., 2011; Loman et al., 2012) translates to millions of incorrect calls per sequenced human genome. Error reduction approaches range from optimizing library preparation techniques to deploying downstream in silico filters (Reumers et al., 2011; Lou et al., 2013).

A Mendelian-inconsistent call (MIC) represents a combination of parent–child trio genotypes at a locus that is in violation of Mendelian inheritance laws with the assumed ploidy. MIC can arise from germline or nongermline de novo mutations, genotype calling errors, or incorrect pedigree information. The de novo mutation rate for humans has been estimated to be ∼1.2e-8 per generation, which translates to an estimated 38 mutations per offspring (Conrad et al., 2011; Kong et al., 2012; Goldmann et al., 2016; Wong et al., 2016). With the rate of true de novo mutations being approximately four orders of magnitude lower than the MIC rate in a representative cohort (Goldmann et al., 2017), most MIC can be attributed to sequencing errors or chromosomal anomalies.

Previous MIC studies were performed on a small number of family trios, with low sequencing depth of coverage, or limited population diversity (Blue et al., 2014; Patel et al., 2014; Pilipenko et al., 2014). Existing tools designed to reduce false-positive calls in family-based sequencing data by checking genotyping calls for consistency (O'Connell and Weeks, 1998; Douglas et al., 2000; Abecasis et al., 2002; Sobel et al., 2002) consider all MIC to be the same and do not consider diverse error mechanisms. Routinely discarding these inconsistencies further increases the bias toward understanding and correcting for errors in regions that are easy to call while excluding regions that can present real insight into diverse error modes (Li et al., 2018). Application of MIC in detecting deletions (McCarroll et al., 2006; Manheimer et al., 2018) and genomic aberrations such as uniparental disomy (Ting et al., 2007; Schroeder et al., 2013) further supports their utility.

We present results to underscore the importance of understanding the origin and characteristics of different types of MIC before discarding them, utilizing them for de novo mutation discovery, or for optimizing variant-calling parameters. We show that incorrect assumptions about the type of MIC can lead to erroneous conclusions. Using an ethnically diverse cohort with 1314 nuclear families, we present an overview of the characteristics of MIC. We highlight that not all MIC can be classified as systematic errors as the inconsistency can be due to limitations in how loci within hemizygous deletions are called. We demonstrate that population-specific MIC frequencies can be used as an additional annotation source for putative de novo mutations to reduce the number of false-positive calls.

## 2. Results

### 2.1. Mendelian-inconsistent signatures and their characteristics

#### 2.1.1. Twelve Mendelian-inconsistent signatures

**FIG. 1.**
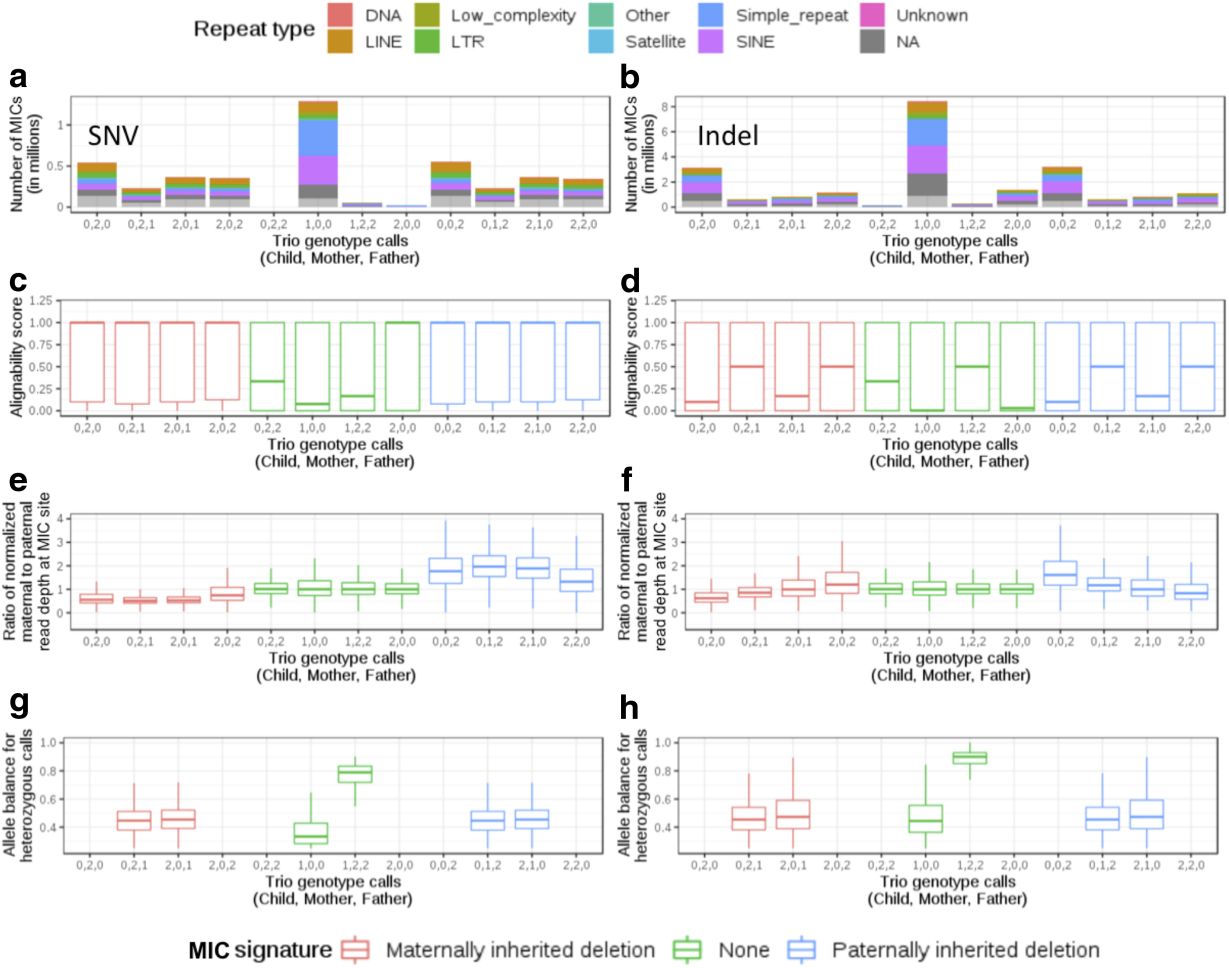
Distribution of raw counts, alignability scores, relative parental depths, and allele balance across 12 Mendelian-inconsistent signatures. **(a, c, e, g)** Represent SNVs while **(b, d, f, h)** display data for Indels. X-axis lists the 12 distinct signatures representing the genotype calls in child, mother, and father where 0, 1, and 2 represent homozygous reference, heterozygous, and homozygous alternate genotypes, respectively. Signatures 1–4 (red) can be found in the event of a maternally inherited allele with a deletion, signatures 5–8 (green) represent absence of a deletion, and signatures 9–12 correspond to paternally inherited allele with a deletion. Repeat type “NA” represents positions that do not overlap with known repeats. SNV, single nucleotide variant; Indel, insertion deletion; MIC, Mendelian-inconsistent call.

**FIG. 2.**
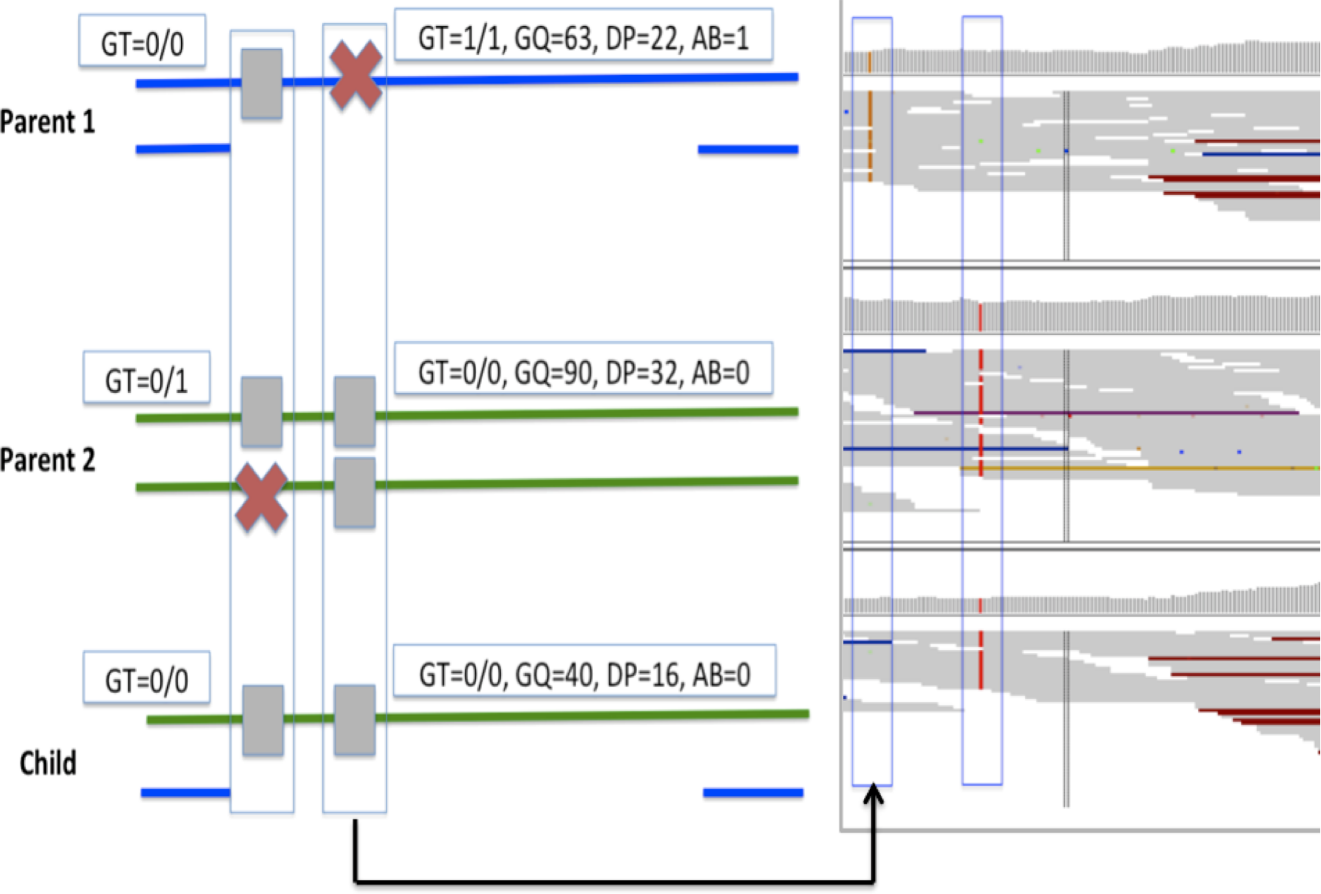
A real example of MIC that overlaps a large deletion and passes quality-based filters. Left panel depicts an MIC with 0,1,2/0,2,1 signature in the trio along with quality metrics for the genotype call in each member of the trio. Parent 1 has a deletion in the bottom allele that is also inherited by the child. Parent 2 does not have the deletion. Panel on the right presents a screenshot of IGV visualization of the alignment for the site and surrounding context. Two MIC in the trio are highlighted within blue rectangles where the first box corresponds to the MIC signature in the left panel. An MIC overlapping the deletion is also illustrated in the figure where Parent 1 and Child are called homozygous reference instead of hemizygous. AB, allele balance; DP, read depth; GQ, genotype quality; GT, genotype; IGV, integrative genomics viewer.

An unphased genotype call in the diploid genome can be homozygous reference (0), heterozygous (1), or homozygous alternate (2). For a given trio, an MIC at a locus can be assigned 1 of 12 signatures based on the genotype calls in child, mother, and father ([0,2,0], [0,2,1], [2,0,1], [2,0,2], [0,2,2], [1,0,0], [1,2,2], [2,0,0], [0,0,2], [0,1,2], [2,1,0], and [2,2,0]) (Fig. 1, X-axes) (Kómár and Kural, 2018). Based on the lengths of reference and alternate alleles against which genotypes are called, an MIC can be a single nucleotide variant (SNV) or an insertion deletion (Indel). These signatures can be further categorized based on properties of MIC within segregating deletions (McCarroll et al., 2006; Ting et al., 2007; Manheimer et al., 2018). When a child inherits a deletion from one of the parents, trio genotype calls within the deletion can manifest as MIC due to hemizygous genotypes being miscalled as homozygous when the locus is assumed to be diploid (Fig. 2). These MIC have distinct genotypes in trio members and can manifest as one of eight signatures based on whether the deletion is maternally or paternally inherited (signatures 1–4 and 9–12; highlighted in red and blue in Fig. 1 for maternally or paternally inherited deletions, respectively) (Manheimer et al., 2018).

We explored other supporting metrics, beyond the genotype calls themselves, which could point to the presence of an underlying deletion. We also evaluated differences in MIC characteristics based on their genomic location, especially when the MIC reside in regions known to be problematic (e.g., known repeats).

#### 2.1.2. MIC in repeats

We first annotated all MIC with features related to sequencing quality to determine if certain MIC types are more likely to result from sequencing errors.

We observed different distributions of raw MIC counts across the 12 signatures for SNVs and Indels (Fig. 1a, b). After filtering low-quality calls (read depth, DP <25% average coverage in sample; genotype quality, GQ <30; allele balance, AB <0.25), 1,863,550 autosomal loci have an MIC in at least one of the trios, with a total of 20,190,613 MIC across all 1314 trios (Table 1). Filtering impacts a larger proportion of MIC that occur at the same position in multiple trios (Supplementary Fig. S1) as frequent MIC are more likely due to systematic issues rather than true biological variation and produce low-quality genotype calls (Conrad et al., 2006; Weir, 2012).

**Table 1.** Overall Mendelian-Inconsistent Call Statistics

*Cohort summary statistics*	
Total No. of errors	20,190,613
Total No. of sites	1,863,550
Total No. of sites with one inconsistent trio	595,218
Average No. of inconsistent trios per MIC site	16 (14–18)

*Trio summary statistics*	
Average % variant sites with MIC	0.63 (0.626–0.633)
Average % unique MIC	1.97 (1.92–2.02)
Average No. of MIC	22,673 (22,539–22,806)
Average No. of unique MIC	453 (440–446)

Ninety-five percent confidence intervals are provided in parentheses when applicable.

MIC, Mendelian-inconsistent call; MIE, Mendelian inheritance error.

**FIG. 3.**
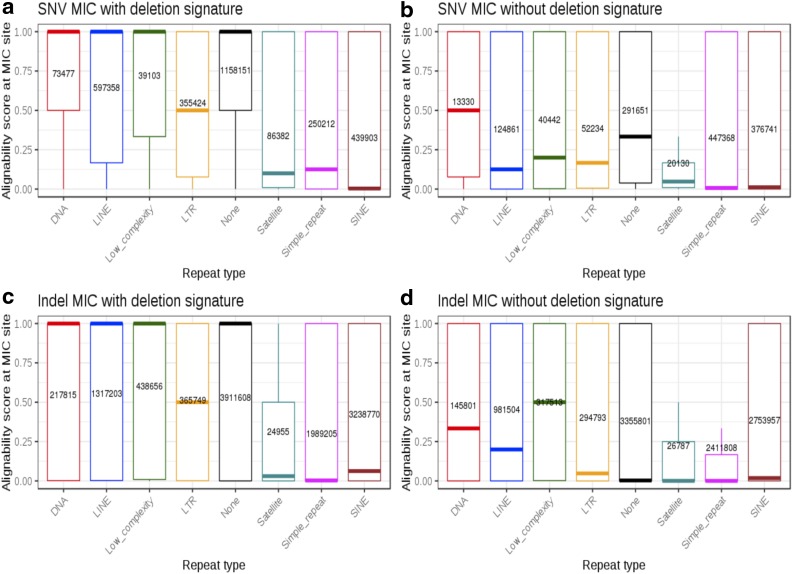
Alignability scores across MIC in different repeat types. Repeat type is shown on X-axis and alignability score at MIC site is on Y-axis. Repeat type “None” represents MIC that do not overlap known repeats. Sample size for each category is displayed at mean alignability score for the category. **(a)** Alignability scores across SNV MIC with the signature of an inherited deletion. **(b)** Alignability scores across SNV MIC without deletion signatures. **(c)** Alignability scores across Indel MIC with deletion signatures. **(d)** Alignability scores across Indel MIC without deletion signatures. LINE, long interspersed nuclear elements; LTR, long terminal repeats; SINE, short interspersed nuclear elements.

Majority of SNV and Indel MIC (both at 67%) overlap with repetitive regions (Fig. 1a, b and Supplementary Table S1), as expected for these error-prone regions. However, there are differences in which type of repeat element each signature is enriched for. The top repeats harboring SNV MIC with deletion signature are long interspersed nuclear elements (LINE) (∼20%) and short interspersed nuclear elements (SINE) (∼15%) (Supplementary Table S1). SINE and simple repeats contain the highest percentage of SNV MIC without deletion signature (27%, 33%) and Indel MIC with (28%, 17%) or without (27%, 23%) inherited deletion signature. To further understand the error mechanism within repeats, we plotted alignability scores for these four MIC categories (SNV MIC with or without deletion signature, and Indel MIC with or without deletion) across different repeat types (Fig. 3). MIC without deletion signature (Fig. 3b, d) are in regions with consistently lower alignability scores when compared with MIC with deletion signature (Fig. 3a, c) for the same repeat type and even for nonrepetitive regions. SINE and simple repeats have the lowest median alignability scores in all MIC categories. In LINE, median alignability scores for SNV and Indel MIC with deletion signature are close to 1. These results imply that MIC in SINE and simple repeats are associated with low alignability and are more likely to be errors. However, MIC with deletion signature that occur in LINE may need to be assessed further before being discarded as errors as they could point to other genomic aberrations such as inherited deletions.

**FIG. 4.**
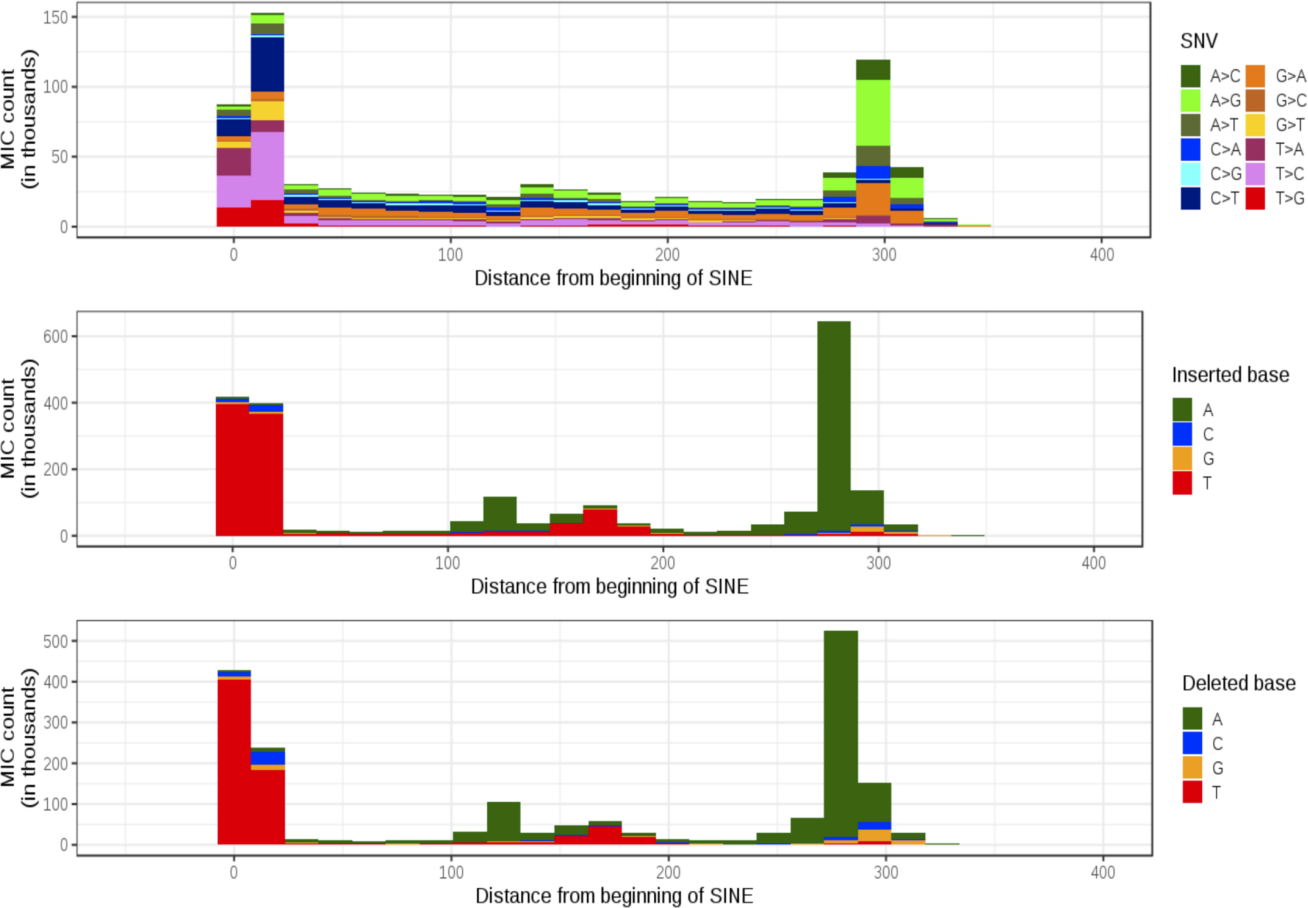
Distribution of SNV and Indel MIC along the length of SINE. X-axis shows the distance of MIC site from beginning of a known SINE, and Y-axis shows the number of MIC at the position across all trios. Color fill represents the SNV, insertion, or deletion as per the legend.

Nearly 60% of Indel MIC occur at sites with single base pair insertion or deletion and SINE contain the largest proportion of these single base pair Indel MIC (Supplementary Fig. S2). SINE also contain the highest percentage (26%) of total MIC across all repeat types (Supplementary Table S1). We analyzed SNV and Indel MIC within SINE to study their distribution along the length of the ∼300 nt long repeat element (Deininger, 2011). SNV and Indel MIC in SINE are concentrated at the beginning and end of the repeat (Fig. 4) and overlap with a poly-T at the beginning of the repeat and a trailing poly-A tail at the end (Supplementary Fig. S3). This is expected as SINE are primarily composed of Alu elements that are ∼300 nt long and contain a poly-T tail in antisense orientation and a poly-A tail in sense orientation (Deininger, 2011). Homopolymer runs are known to be enriched for polymerase chain reaction (PCR) errors and therefore contain a high number of MIC (Li, 2014). We can conclude from these findings that poly-A and poly-T runs contribute to MIC enrichment within SINE and these inconsistencies have a high likelihood of being due to PCR errors instead of deletions or true de novo events.

#### 2.1.3. Alignability score, ratio of parental read depths, and allele balance

SNV MIC overlapping a hemizygous deletion (red and blue signatures in Fig. 1c) have higher alignability scores compared with other categories of MIC and are not limited to regions that are known to be difficult to align (mean alignability score of 0.6 vs. 0.4; *t*-test *p*-value <2 × 10^−16^) (Supplementary Fig. S4). Therefore, filtering on alignability score will reduce a larger proportion of SNV MIC without deletion signature compared with those with a deletion. Among the 12 signatures, the highest number of MIC is attributed to the 1,0,0 signature for child, mother, and father genotypes (Fig. 1c), which is the most frequent nondeletion signature (counts displayed in Fig. 1a) and represents trio genotype calls when child has an alternate allele not found in either parent. This signature also has the lowest median alignability score for both SNVs and Indels. These observations highlight the challenges associated with distinguishing true de novo events from false positives as most studies consider 1,0,0 MIC as the initial set of candidate de novo mutations (Li et al., 2012; Neale et al., 2012).

The ratio of normalized maternal to paternal read depth also differs between SNV MIC with or without an inherited deletion (Fig. 1e, f). The median ratio is closer to 0.5 for maternal deletions and closer to 2 for paternal deletions (Supplementary Fig. S5). The observation is expected if we consider that an MIC due to a hemizygous deletion inherited from the mother should correspond to a maternal read depth that is close to half of the paternal read depth at that locus if the father does not have the deletion. However, the trend is not as evident in MIC with 2,0,2 or 2,2,0 genotypes as they include cases where both parents could have a deletion, leading to a ratio of maternal to paternal depth closer to 1. These results support using differences in parental read depths as an additional criterion for selecting SNV MIC for application in detection of deletions and for filtering de novo mutations.

Allele balance is close to 0.5 for heterozygous calls in all signatures for inherited deletion (mean 0.46; 95% confidence interval width <0.005), whereas the median allele balance for 1,0,0 signature is close to 0.3 (Fig. 1g, h). Among the four MIC categories (SNV MIC with or without deletion signature, and Indel MIC with or without deletion signature) SNV MIC with deletion have the highest percentage (38%) of calls in non-repetitive regions (21%, 34%, and 32% for SNV calls without deletion signature, Indels with, and Indels without deletion signature, respectively). Therefore, a higher proportion of these deletion-specific SNV MIC will be immune to filters based on overlap with a repeat, alignability scores, and allele balance but will be impacted if differences in read depth in trio members are also considered. The result is highly relevant for studies that consider signatures other than 1,0,0 to be candidate de novo events, thereby waiving a restriction imposed to limit false positives (Li et al., 2012; Neale et al., 2012; Wong et al., 2016).

We selected a trio to evaluate our findings on different error mechanisms in different repeat types, and the impact of using alignability score and ratio of parental read depths for filtering. We extracted all SNV MIC with putative maternally inherited deletion signatures within LINE that had an alignability score of 1 and ratio of normalized maternal to paternal read depth less than 0.5. This resulted in 31 MIC. Of these, we could confirm 25 MIC (81%) to be within maternally inherited deletions with defined breakpoints occurring in different regions of the genome (example in Supplementary Fig. 6a) by visually inspecting the alignments in integrative genomics viewer (IGV) (Robinson et al., 2011). However, using the same criteria on MIC within SINE resulted in only four candidates as few MIC in SINE occur in regions of high alignability. All four MIC in SINE are errors and reside within soft-clipped reads (example in Supplementary Fig. 6b).

### 2.2. Mendelian-inconsistent SNV calls with deletion signature are population-specific

**FIG. 5.**
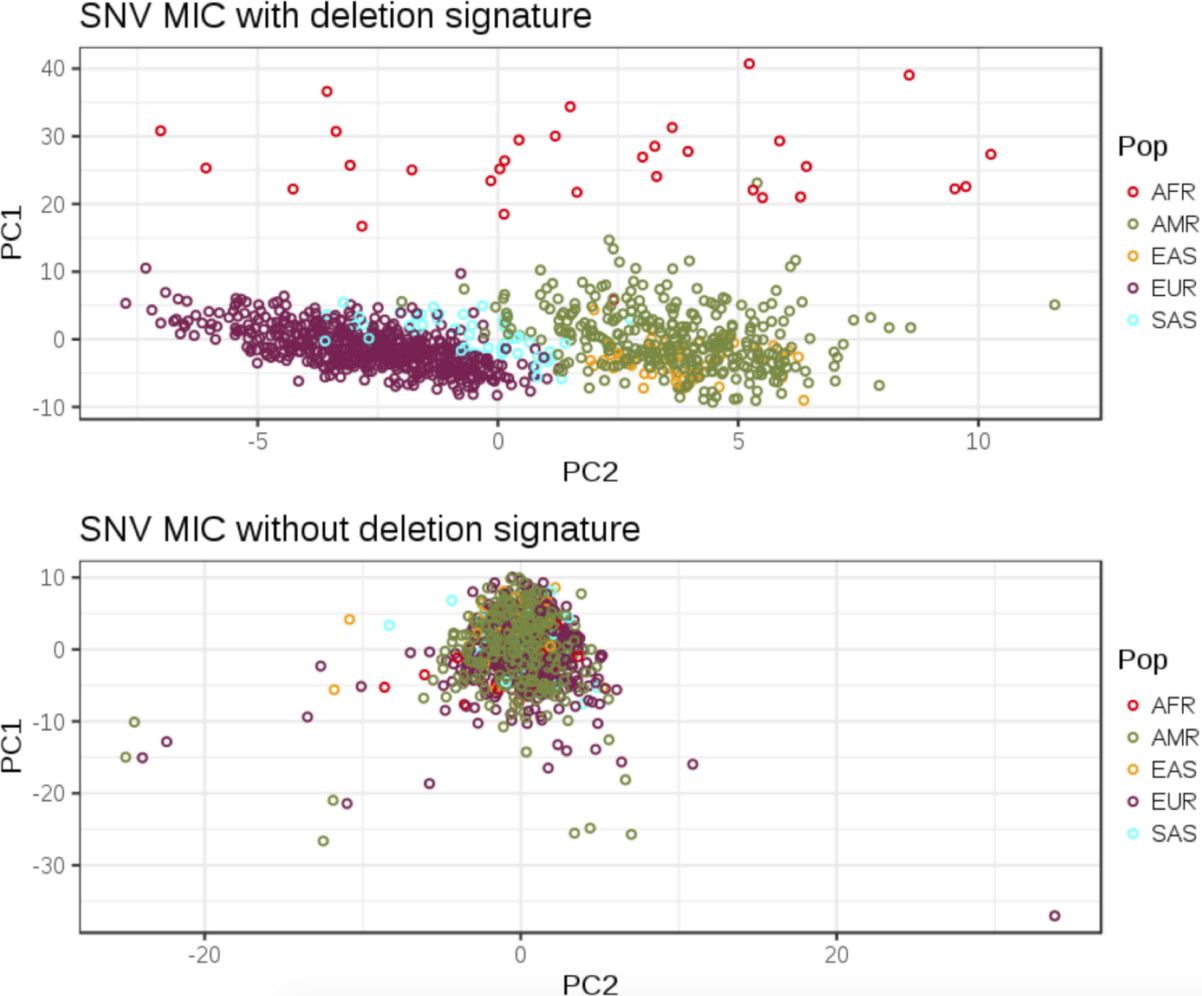
PCA plots obtained with total MIC with or without deletion signatures in 1 Mb genomic windows in trios. Points are colored by calculated ancestry. X-axis and Y-axis denote PC2 and PC1, respectively. PC1, first principal component; PC2, second principal component; PCA, principal component analysis.

SNV MIC have been used for detection of deletions (McCarroll et al., 2006; Manheimer et al., 2018), but we wanted to extend their application to detect putative population-specific deletions. We performed principal component analysis (PCA) on SNV MIC with and without deletion signatures (Fig. 5). We aggregated MIC counts (with or without deletion-specific signatures) in 1 Mb window for each trio across all the autosomes as MIC cluster in regions with a hemizygously inherited deletion (McCarroll et al., 2006; Manheimer et al., 2018). The first principal component (PC1) correlates with the number of MIC in a given trio (Supplementary Fig. S7). The second principal component (PC2) could stratify the major populations with deletion-specific MIC but not for nondeletion MIC.

The 1 Mb windows with the highest PC2 loadings (Supplementary Fig. S8) and enrichment for MIC with deletion signatures in AMR (Admixed American) trios were found in regions known to be associated with body mass index or obesity in Hispanic population. 1p36 is known to be associated with serum ghrelin and obesity-related phenotypes in Hispanic children (Voruganti et al., 2007). The other regions associated with obesity are 16q12.2 (Wu et al., 2002), 3q29 (Kettunen et al., 2009), 2p22.3, 2q14.3, 6q23.3, and 8p11.23 (Yang et al., 2007). Additionally, 1p36.23 and 1p34.2 are associated with gall bladder cancer (Puppala et al., 2006) and 17q25.1 with age of onset in late-onset Alzheimer's disease in Caribbean Hispanics (Lee et al., 2008). An example of a population-specific 3q29 deletion enriched in AMR is shown in Supplementary Figure S9. We are further investigating these regions for AMR-specific deletions and their contribution to obesity-related phenotypes in our cohort.

We have created bedGraph files for each population to summarize population-specific counts at every locus where an MIC is found in at least one trio. These are provided as Supplementary Materials in an accompanying zipped archive. The bed files can be loaded as tracks and viewed in UCSC Genome Browser (Kent et al., 2002).

### 2.3. A practical application: investigating de novo mutations in denovo-db

**FIG. 6.**
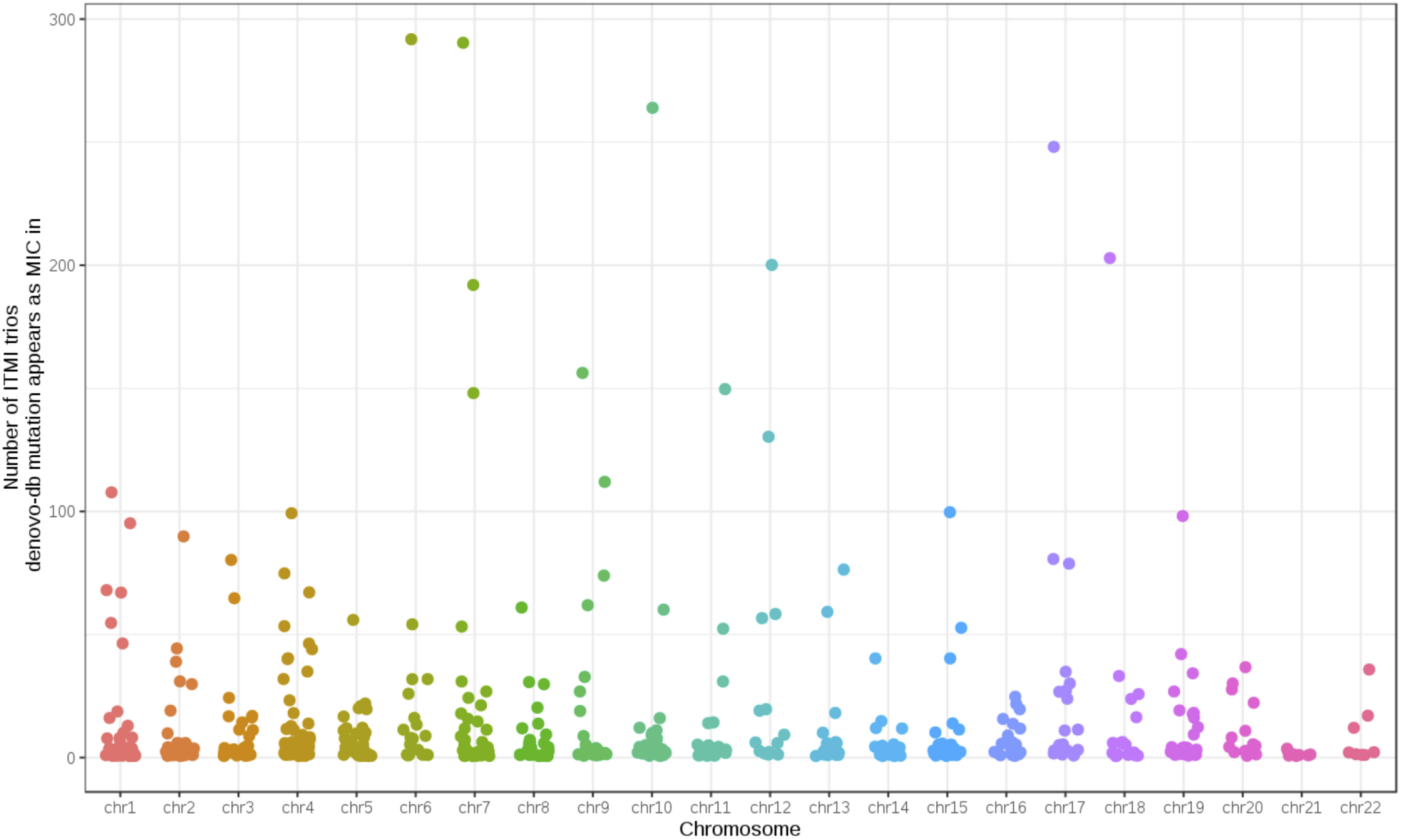
Frequency of occurrence for each de novo mutation from denovo-db that overlaps with an MIC in at least one ITMI trio. Each point denotes one de novo mutation site. Points are colored by the autosome. ITMI, Inova Translational Medicine Institute.

To demonstrate the utility of MIC bedGraphs and emphasize the need for annotating candidate de novo mutations with MIC frequencies in a larger population, we utilized data from denovo-db, which is a database of germline de novo mutations in the human genome (Turner et al., 2017). We excluded samples from the Simons Simplex Collection due to restricted data usage. There are 415,515 de novo mutations across 270,506 unique chromosomal positions and 11,518 samples. Of these, 955 loci with a de novo mutation also have an MIC in at least 1 ITMI (Inova Translational Medicine Institute) trio. These overlapping entries span 577 unique chromosomal positions, 611 samples from denovo-db, and 1054 ITMI trios. Figure 6 shows the number of ITMI trios each overlapping de novo mutation is observed in across the autosomes. Supplementary Figure S10 displays the number of denovo-db samples overlapping ITMI MIC appear as de novo mutations in. Because true de novo mutations are expected to be rare, with low recurrence rate, those mutations overlapping MIC sites in multiple ITMI trios are likely to result from systematic sequencing errors. This suggests that MIC frequencies from the ITMI trios could be used for assigning confidence levels to candidate de novo mutations.

**FIG. 7.**
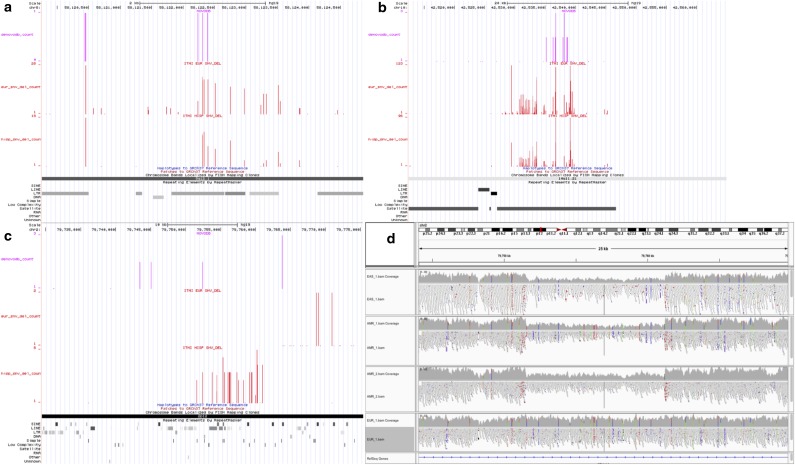
Examples of overlapping sites between de novo mutations in denovo-db and MIC in ITMI trios. Tracks are from bedGraph files loaded into UCSC Genome Browser. The top track (pink) is for denovo-db entries where height of the bar represents the number of entries for the de novo mutation. The bottom two tracks (red) are for MIC in ITMI trios from EUR and AMR ancestry, respectively. The height of each bar represents the number of trios an inconsistent call was found in. Repeat element tracks are included at the bottom. **(a)** An example of denovo-db sites overlapping with MIC in multiple ITMI trios and within a long terminal repeat. **(b)** An example of a denovo-db site in satellite DNA of chromosome 10 that is Mendelian-inconsistent in >250 ITMI trios. **(c)** A denovo-db site in chromosome 2 overlapping an AMR-specific deletion. **(d)** A view of the alignment in chromosome 2 with an AMR-specific deletion (top three tracks). The region is enriched for MIC in AMR as shown in the third track (red) in **(c)**. AMR, Admixed American; EUR, European.

We selected a few representative examples from the overlapping mutations to outline the application of the population-specific ITMI bedGraphs (Fig. 7). In the first example by visualizing denovo-db sites (pink track in Fig. 7a) against ITMI MIC in EUR (European) and AMR trios (red tracks), we can see that the putative de novo mutations are at exact positions that overlap with MIC in multiple ITMI trios and are within a long terminal repeat. EUR and AMR have the highest representation in our cohort and are, therefore, selected for these examples. Figure 7b shows a chromosome 10 region overlapping satellite DNA with inconsistent calls in >250 trios for one of the denovo-db sites. Figure 7c depicts a denovo-db mutation overlapping a chromosome 2 region with an AMR-specific deletion (Fig. 7d). These examples highlight the need for rigorous inspection of putative de novo mutations especially when candidate de novo events are not limited to loci where the parents are called homozygous for the reference allele and the child is called heterozygous, an approach adopted to reduce false positives (Li et al., 2012; Neale et al., 2012). The accompanying bedGraphs are useful when prioritizing de novo mutation candidates in non-EUR trios as it is a unique resource for assessing if a region is enriched for MIC due to population-specific effects.

## 3. Discussion

The utility of considering familial relationships for error estimation and correction during detection of variants from sequencing data has been demonstrated before (Ting et al., 2007; Roach et al., 2010; Chen et al., 2013; Martin et al., 2014; Kómár and Kural, 2018). In trio-based studies, MIC are often used for estimating sequencing error rates and for optimizing filtering parameters. However, MIC are treated uniformly as errors and variant calling and filtering pipelines are optimized with the goal of minimizing their occurrence. Our results demonstrate that different types of MIC exist and exhibit distinct characteristics. We employed WGS data from 1314 trios from diverse populations to understand the characteristics and utility of MIC and highlight the need to exercise caution while selecting MIC for application in variant calling optimization or de novo mutation detection.

The repetitive nature of the human genome has been known to introduce mapping and alignment challenges (MacArthur et al., 2012; Treangen and Salzberg, 2012). Recent work has highlighted that benchmark data sets such as GIAB (Genome In A Bottle) (Zook et al., 2014) and PlatGen (Platinum Genome) (Eberle et al., 2017) are biased toward genomic regions that are easy to sequence and call (Li et al., 2018). Our work is a step toward understanding error modes in repeats and regions that are difficult to align and call. We observed highest MIC density in chromosome 19 (Supplementary Table S2), which has been studied previously due to its high repeat density (Grimwood et al., 2004). We found that MIC are enriched in SINE due to the presence of poly-A and poly-T stretches. SINE are 100–300 nt long and have a genomic coverage of 15%, whereas LINE are 500–8000 nt long and cover 21% of the genome, which means that a higher number of SINE are found in the genome compared with LINE (1.8 million vs. 1.5 million) (Treangen and Salzberg, 2012). Alu elements are primate-specific SINE, which are ∼300 nt in length, and propagate within a genome through retrotransposition. The abundance of Alu elements combined with their structure and presence of homopolymer runs of variable length poses sequencing, mapping, and variant calling challenges (Lander et al., 2001; Deininger, 2011). SINE contained the highest percentage of MIC among all repeat types (26%) (Supplementary Table S1), and these MIC were associated with lower alignability scores compared with deletion-specific MIC in LINE. Our results show that MIC in SINE are likely to be errors, whereas MIC with deletion signature that occur in LINE could point to genomic aberrations.

We corroborated previous findings that MIC are enriched in repeat elements and quality metrics vary between Mendelian-consistent and Mendelian-inconsistent calls (Blackburn et al., 2014; Martin et al., 2014; Patel et al., 2014; Pilipenko et al., 2014) but extended these results to describe different types of MIC. Quality-based filtering has a greater impact on MIC that occur across a higher proportion of trios compared with unique MIC found in a single trio at a site. MIC can have 12 distinct signatures based on the genotype calls in child and parents. Eight of these signatures can correspond to a deletion inherited by the child from one of the parents.

Origin and properties of MIC signatures need to be considered while modeling filters for de novo mutation calling. For example, a previous study estimates callable sites for de novo mutations by assuming that only de novo events can lead to Mendelian violations where one parent is homozygous reference at a locus, the other is homozygous alternate, and the child is not a heterozygote (Besenbacher et al., 2015). However, such violations can be caused by inherited deletions instead of a true de novo event if a diploid call is made within a hemizygous deletion. The same study also estimates a higher mutation rate per generation compared with previous studies (Kong et al., 2012; Michaelson et al., 2012) and warrants the question if modifying the assumption around the origin of these MIC would lead to a different estimate of mutation rate. Our results show that properties such as alignability scores, ratio of parental read depths, and allele balance vary among the 12 signatures for SNV and Indel calls. Therefore, filters based on these criteria have varying impact on different MIC signatures.

The above results highlight the importance of considering the type and location of MIC while using these inconsistent calls for benchmarking pipelines. For example, if pipeline A has an overall lower number of MIC compared with pipeline B due to deletion-specific MIC in LINE or regions with no repeats that are exclusive to the latter, it cannot be directly inferred that pipeline A performs better. The genotype calls need to be assessed to confirm that pipeline B is not, in fact, flagging true biological variation (e.g., inherited deletions) that pipeline A is ignoring.

Using MIC enrichment across populations, we found that the top regions with different MIC profiles in EUR and AMR trios are known to be associated with obesity in Hispanic population. These regions are 1p36 (Voruganti et al., 2007), 16q12.2 (Wu et al., 2002), 3q29 (Kettunen et al., 2009), 2p22.3, 2q14.3, 6q23.3, and 8p11.23 (Yang et al., 2007). However, while it has been suggested that Mexican lineage could have a genetic basis for being a risk factor for obesity, it is difficult to distinguish between the contribution of cultural and genetic factors to disparities in weight (Liu et al., 2015). We are using these results as the basis for subsequent analysis, which includes confirming if these loci contain AMR-specific deletions, and assessing if there is association between these deletions and obesity-related phenotypes.

Our analysis has limitations that require discussion. Further work is required to develop and optimize quality-based filters using MIC data for benchmarking. Detection of deletions with clustered MIC has limitations in that the presence of a heterozygous deletion has to overlap with SNVs to lead to MIC in the region. A future goal is to assess the impact on MIC of mapping to GRCh38. We expect joint trio-based variant calling to reduce the number of MIC but aim to compare the proportion of calls overlapping deletions that are converted to missing, reported as hemizygous, or assigned low quality.

Our results indicate that MIC characteristics need to be considered when selecting them for different applications. Clustered MIC and MIC with the signature of calls overlapping putative deletions should be evaluated further to ensure these are not in reality hemizygous calls due to an overlapping deletion. Additionally, Mendelian-consistent calls within a cluster of MICs also need to be flagged and inspected before allele frequencies and admixture coefficients as these could be hemizygous calls that do not manifest as Mendelian inconsistencies, but the genotypes could still be incorrect. We analyzed de novo mutations in denovo-db (Turner et al., 2017) that overlap with MIC in our trios and provided examples of how ITMI MIC bedGraphs can be used to flag de novo mutations that require further inspection before being reported. Also, our results highlight the importance of including individual parent and child genotype calls and ancestry information when reporting de novo mutations. A de novo mutation with a deletion signature should be further inspected to rule out an inherited deletion. The bedGraphs can be a useful resource for annotating candidate de novo mutations with population-specific frequencies, especially when non-EUR trios are being studied.

## 4. Methods

### 4.1. Sequencing data

The 1314 family trios (father, mother, and child) with validated pedigrees were obtained from an Institutional Review Board-approved childhood longitudinal WGS study. Informed consent was obtained for all subjects in the study. Whole blood samples were collected and genomic DNA was extracted (Bodian et al., 2014). Samples were sent to Illumina (San Diego, CA) where they were sequenced at >40 × coverage with the Illumina Whole Human Genome Sequencing Service Informatics Pipeline version 2.01-03 (https://github.com/sequencing) and mapped to the hg19 human reference genome (Lander et al., 2001). Quality control was performed, which included checking relatedness among samples using Ancestry and Kinship Toolkit (AKT) (Arthur et al., 2017), and samples suspected to be swapped were excluded from the analysis.

### 4.2. Quality metrics and analysis in known repeats

Individual genomic Variant Call Format (gVCF) files from all subjects were combined using agg (Illumina, 2015). Resulting merged Variant Call Format (VCF) file was then processed to generate a list of MIC in the autosomes using bcftools (version 1.3) (Danecek et al., 2011) mendelian plugin and custom shell scripts (Supplementary Fig. S11). We required genotype calls to have a read depth DP ≥25% of the average coverage, allele balance AB ≥0.25 (for heterozygous calls), and quality score GQ ≥30 to exclude calls with very low quality. MIC-bearing loci were annotated with annovar (Wang et al., 2010). RepeatMasker (Smit et al., 1996) track from UCSC Table Browser (Kent et al., 2002) was used for annotating sites overlapping repeat elements. Offset from the beginning of the SINE and sequence context of MIC positions with SINE were computed using BEDtools (Quinlan and Hall, 2010). Ratio of normalized parental read depth for any MIC in a trio was calculated as the ratio of maternal read depth as a proportion of average coverage for the maternal sample to paternal read depth as a proportion of average coverage for paternal sample at the site of MIC. Alignability scores (36 mers) were downloaded as CRG alignability tracks from UCSC Table Browser (Karolchik et al., 2004) (downloaded on October 12, 2018). Genotype quality, allele balance, and ratio of parental read depths for the 12 MIC signatures were extracted using a combination of bcftools and custom R (version 3.4.0) scripts. R package ggplot2 (version 2.2.1; https://github.com/tidyverse/ggplot2) was used for creating plots.

### 4.3. Population-specific MIC tracks

To assign each individual to an ancestral population, the samples were projected onto the 1000 genomes project (1000 Genomes Project Consortium et al., 2015) principal components using the 17,535 SNPs specified in AKT git repository using AKT (Arthur et al., 2017). The projections were then clustered into five clusters with PCs 1–3 using AKT. The clusters were defined by the five 1000 genomes phase 3 super populations (namely AFR, AMR, EAS, EUR, and SAS). Those samples with silhouette score of >0.6 were assigned the ancestry that the cluster belongs to, whereas others are assigned “OTHERS” as their ancestry. For population-specific analyses, we created groups to include all trios where both parents are assigned the same ancestry. This resulted in 545 EUR, 316 AMR, 55 EAS (East Asian), 49 SAS (South Asian), 29 AFR (African), and 22 OTHERS trios. These groupings were then used to generate population-specific bedGraphs (www.genome.ucsc.edu/FAQ/FAQformat.html#format1.8) where the bedGraph file for a population lists all chromosomal positions where an MIC is detected in at least one trio in addition to the total number of trios from the population that have an MIC at the position.

### 4.4. PCA and detection of regions with population-specific MIC enrichment

We generated 1 Mb genomic windows using BEDtools makewindows (Quinlan and Hall, 2010) and calculated the total number of each type of MIC in a given 1 Mb for all trios. We used these aggregated MIC counts for each trio to perform PCA with the R package pcaMethods (Stacklies et al., 2007) for SNV MIC with or without genotype signatures corresponding to an inherited deletion (Fig. 3). PC2 loadings were obtained and top 1 Mb windows influencing the projection were extracted. Alignments in putative population-specific deletions were viewed in IGV (Robinson et al., 2011).

## 5. Conclusions

In the current research, it was demonstrated that different types of MIC exhibit different properties depending on the cause of the inconsistency. MIC are clustered within germline deletions and are enriched in SINE. ITMI MIC bedGraphs can be used for annotating candidate de novo mutations with population-specific frequencies and flagging calls that could be due to inherited deletions or repeats. In conclusion, Mendelian inconsistencies and other types of errors need to be characterized better before being discarded as they could be representative of an underlying feature of the data and could contribute toward a better understanding of broader error mechanisms.

## Ethics Approval and Consent to Participate

Whole-genome sequences are from Institutional Review Board-approved First 1,000 Days of Life Study.

## Consent for Publication

Not applicable. The article does not contain any individual person's data.

## Availability of Data and Materials

BED files summarizing MIC sites and associated MIC rates for each population are provided as Supplementary Materials. The files can be loaded as tracks and viewed in UCSC Genome Browser.

## Supplementary Material

Supplemental data
