# Supplementary material for: Mendelian Inconsistent Signatures from 1314 Ancestrally Diverse Family Trios Distinguish Biological Variation from Sequencing Error
Source: J Comput Biol. 2019 May 8;26(5):405–19. doi: 10.1089/cmb.2018.0253 (PMC6533806; doi:10.1089/cmb.2018.0253)
Supplement: Supplemental data [file Supp_Data.zip › Supp_Table1.docx]

Supplementary Table S1. Distribution of Mendelian-Inconsistent Call Counts Across Different Repeat Types for Single Nucleotide Variants and Indels

**
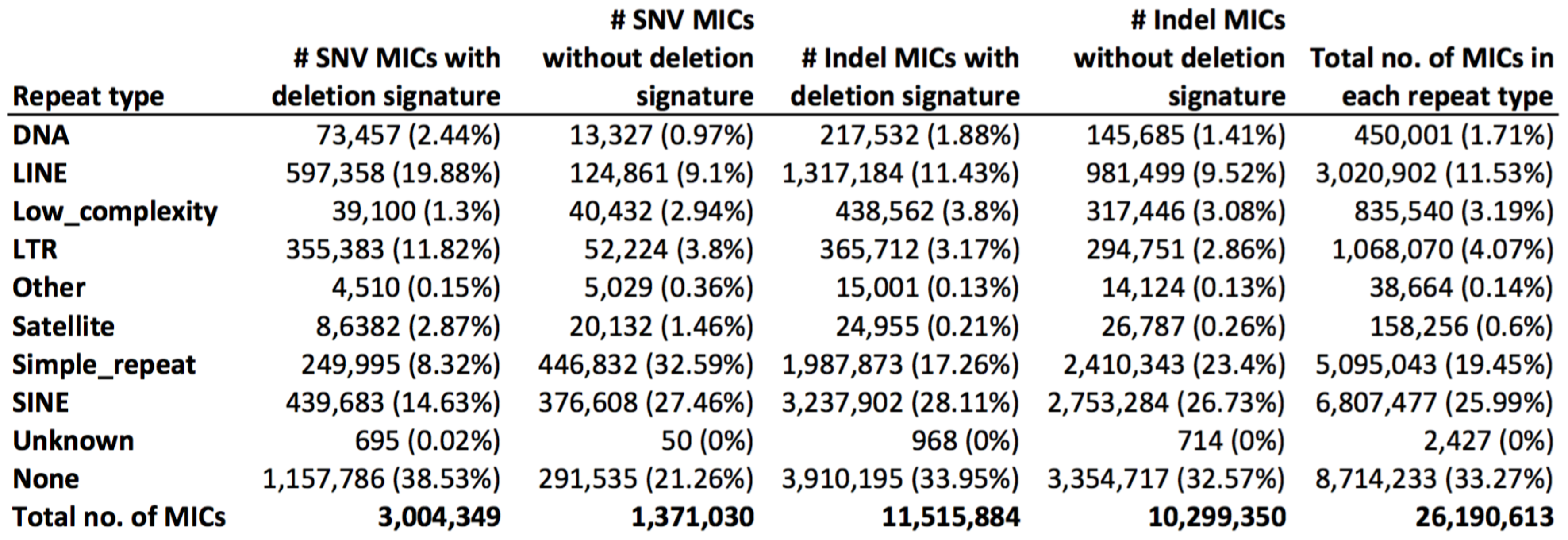
**
