# Supplementary material for: Mendelian Inconsistent Signatures from 1314 Ancestrally Diverse Family Trios Distinguish Biological Variation from Sequencing Error
Source: J Comput Biol. 2019 May 8;26(5):405–19. doi: 10.1089/cmb.2018.0253 (PMC6533806; doi:10.1089/cmb.2018.0253)
Supplement: Supplemental data [file Supp_Data.zip › Supp_Data.pdf]

Supplementary Material

Supplementary Table S1. Distribution of Mendelian-Inconsistent Call Counts Across Different Repeat Types for Single Nucleotide Variants and Indels

Supplementary Table S2. Overall Mendelian-Inconsistent Call Statistics for Each Autosome

Autosome	*Variants ($\times 10^6$)*	*MIC sites*	*No. of MIC sites per 1000 variant sites*	*MIC sites overlapping repeats*	*% MIC sites overlapping repeats*	*No. of MIC sites per 1 kb*
chr1	13.21	305,810	23.15	225,039	73.59	1.23
chr2	13.51	290,446	21.50	213,961	73.67	1.19
chr3	10.29	231,046	22.45	179,292	77.60	1.17
chr4	10.48	258,814	24.70	199,532	77.09	1.35
chr5	10.39	217,360	20.92	166,580	76.64	1.20
chr6	9.05	235,084	25.97	177,011	75.30	1.37
chr7	10.08	237,449	23.56	172,838	72.79	1.49
chr8	8.75	201,867	23.07	144,497	71.58	1.38
chr9	10.71	233,368	21.79	161,262	69.10	1.65
chr10	8.42	188,857	22.44	135,697	71.85	1.39
chr11	7.50	180,554	24.08	138,832	76.89	1.34
chr12	6.89	170,713	24.77	131,946	77.29	1.28
chr13	4.77	117,183	24.56	85,392	72.87	1.02
chr14	5.08	129,636	25.54	93,466	72.10	1.21
chr15	6.23	141,241	22.68	94,671	67.03	1.38
chr16	6.09	137,433	22.56	95,424	69.43	1.52
chr17	4.62	124,768	27.03	88,445	70.89	1.54
chr18	3.78	87,674	23.22	64,659	73.75	1.12
chr19	3.66	133,308	36.43	99,930	74.96	2.25
chr20	3.03	75,515	24.89	55,728	73.80	1.20
chr21	1.88	54,204	28.80	38,221	70.51	1.13
chr22	2.67	77,129	28.90	49,101	63.66	1.50

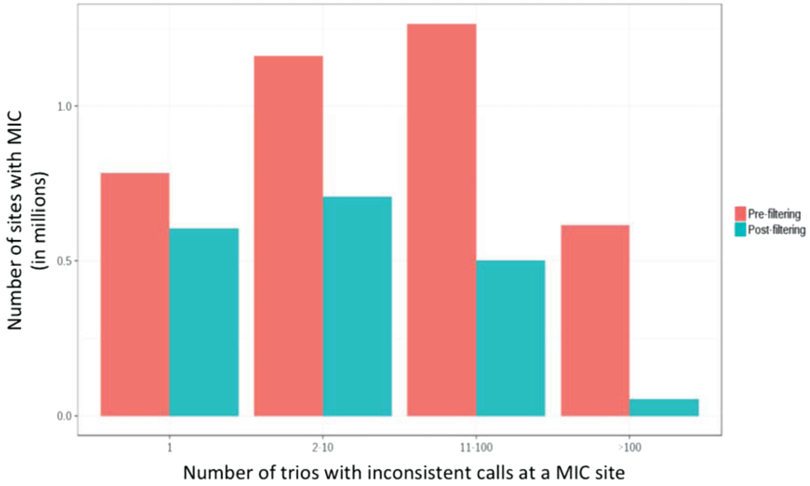

SUPPLEMENTARY FIG. S1. Pre- and post-filtering distribution of number of Mendelian-inconsistent trios at MIC sites. X-axis contains four categories for number of Mendelian-inconsistent trios at any given MIC site before and after filtering. The first category denotes number of sites with a unique MIC (inconsistency in a single trio out of a total of 1314 trios). Y-axis contains the number of MIC sites that have inconsistencies in a given number of trios corresponding to the bin on X-axis. MIC, Mendelian-inconsistent call.

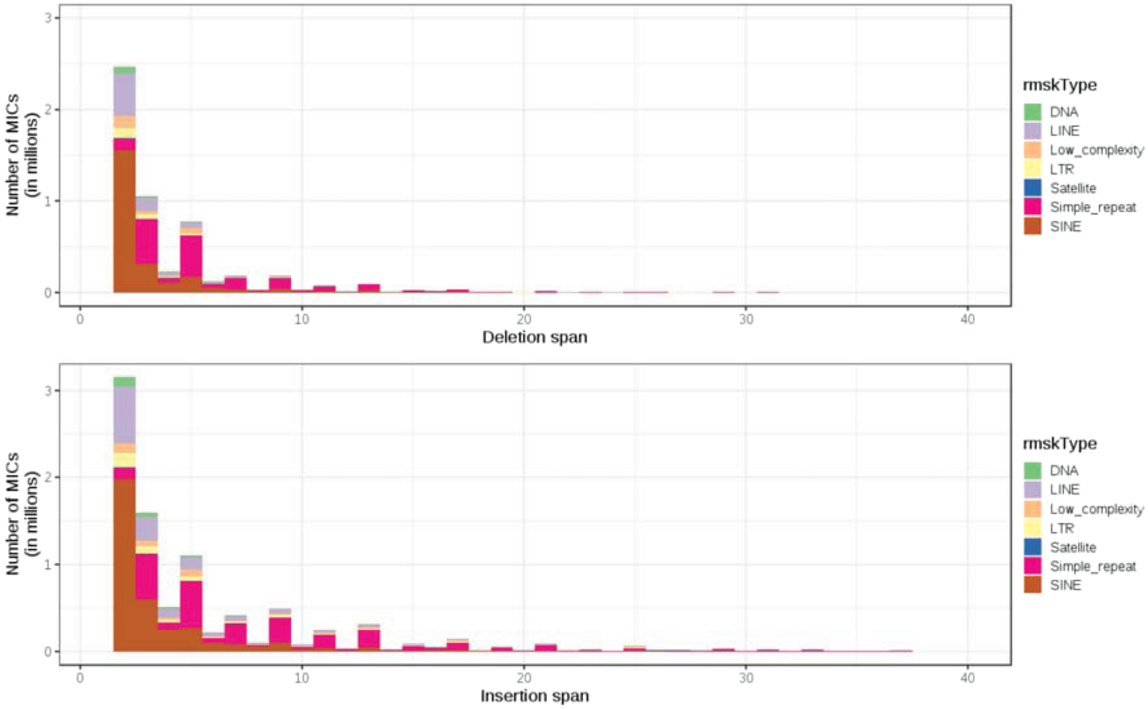

SUPPLEMENTARY FIG. S2. Distribution of length of deletion or insertion at sites with MIC within repeats. Color fill represents the repeat type in which MIC occurs.

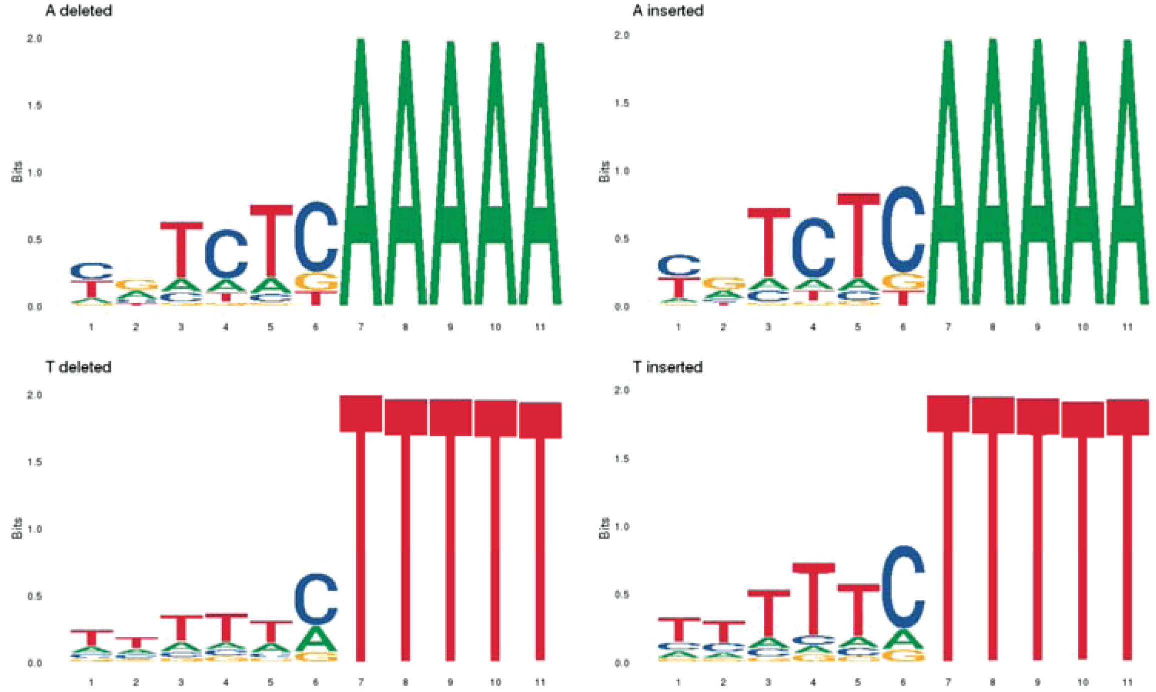

SUPPLEMENTARY FIG. S3. Sequence context for deleted A and T bases within SINE. SINE, short interspersed nuclear elements.

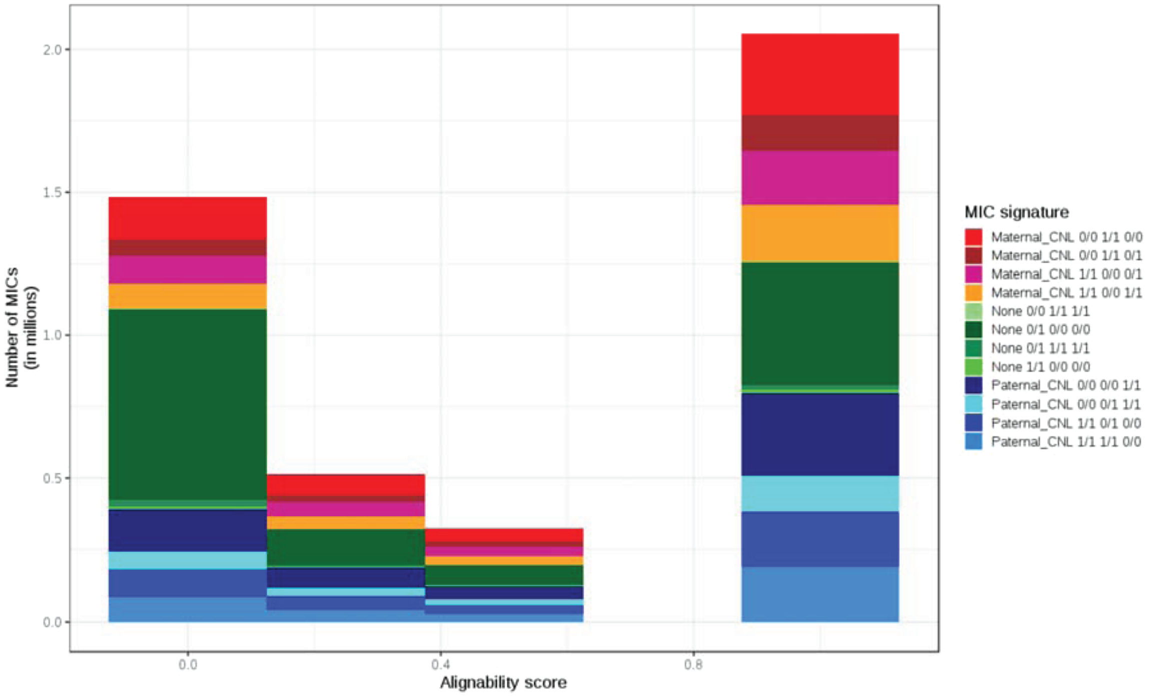

SUPPLEMENTARY FIG. S4. Histogram of alignability scores across the 12 signatures for SNV MIC. Color fill represents the type of MIC signature (with maternal deletion, no deletion, or paternal deletion). SNV, single nucleotide variant.

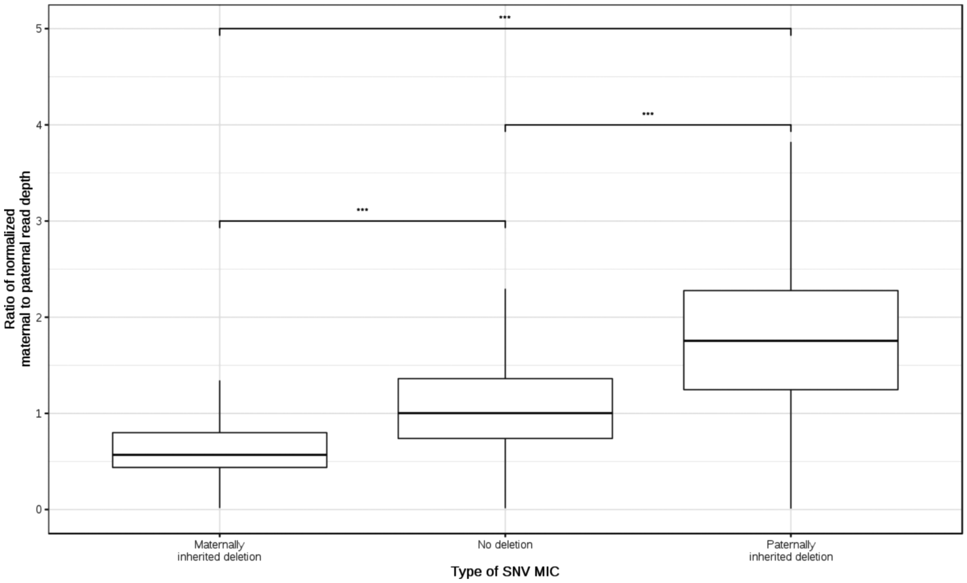

SUPPLEMENTARY FIG. S5. Ratio of normalized maternal to paternal read depth for SNV MIC with or without deletion. ***indicates a *p*-value $<1 \times 10^{-5}$.

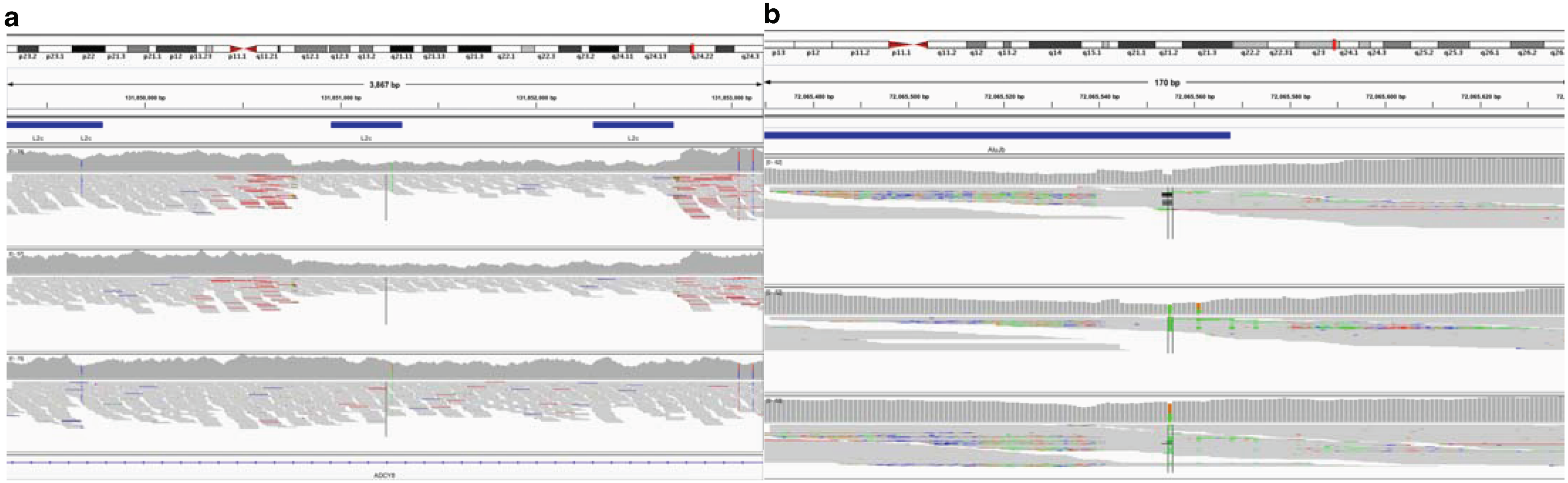

SUPPLEMENTARY FIG. S6. Example of MIC within LINE and SINE. **(a)** Shows an example of an MIC within LINE that overlaps a deletion in child (top track) inherited from the mother (middle track). **(b)** Shows an MIC in SINE poly-A tail and residing within soft-clipped reads obtained using the same filtering criteria as those used for LINE. LINE, long interspersed nuclear elements.

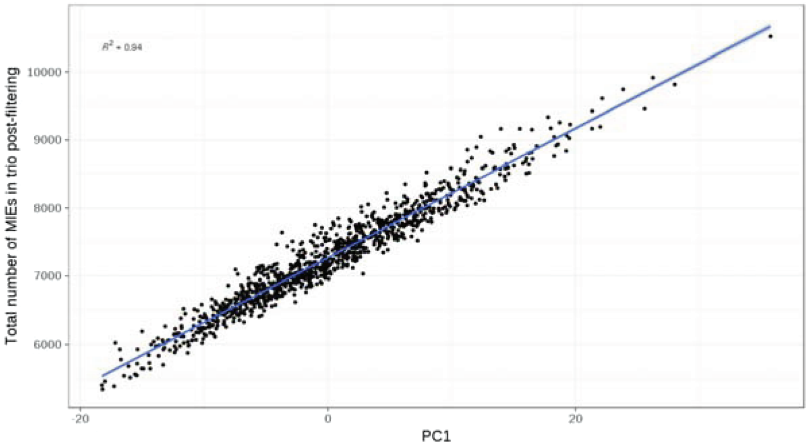

SUPPLEMENTARY FIG. S7. PC1 for a given trio correlates with the number of MIC in the trio for SNV MIC with or without deletion signatures. PC1, first principal component.

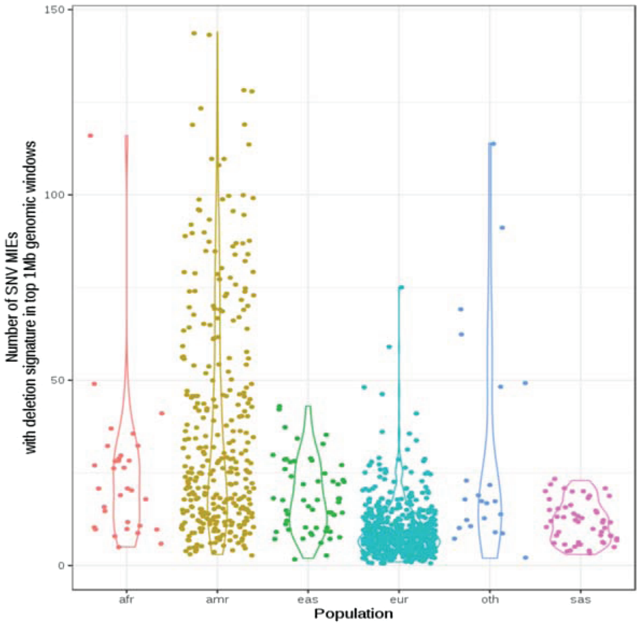

SUPPLEMENTARY FIG. S8. Population-specific distribution of SNV MIC with deletion signature in 1 Mb windows with highest PC2 loadings. PC2, second principal component.

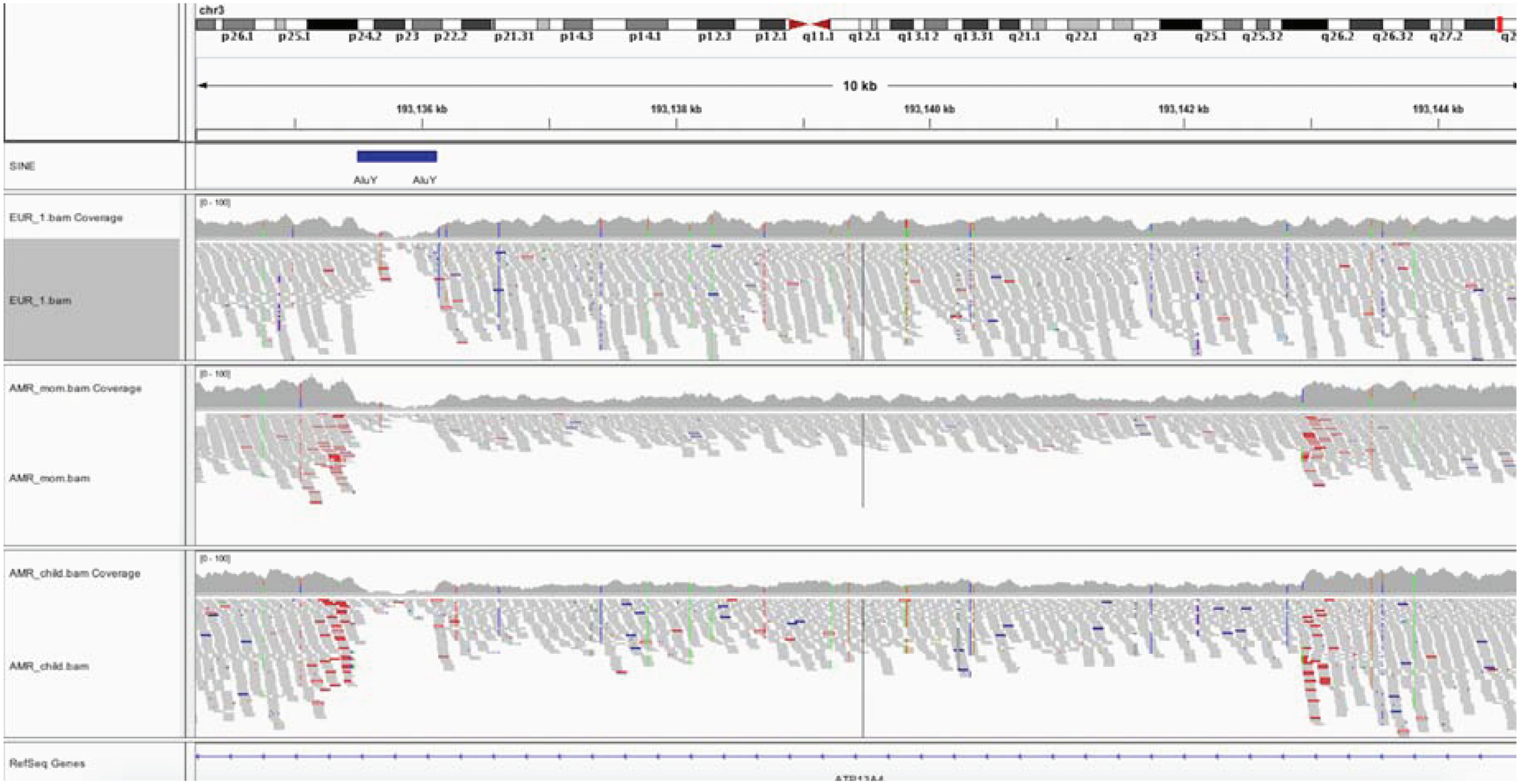

SUPPLEMENTARY FIG. S9. A deletion in 3q29 corresponding to an enrichment for MIC in AMR trios. The region has the highest loading for PC2. The top track is for a EUR subject who does not have the deletion. The bottom two are for AMR subjects and show a deletion with defined breakpoints. AMR, Admixed American; EUR, European.

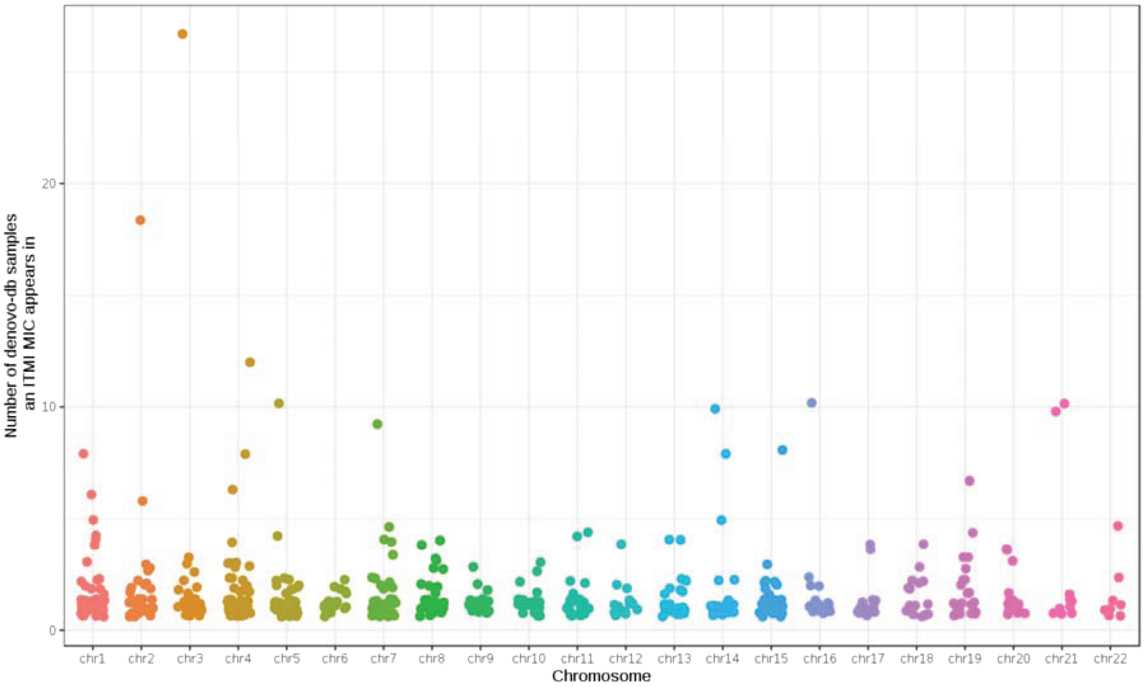

SUPPLEMENTARY FIG. S10. Frequency of occurrence in ITMI trios for each overlapping de novo mutation from denovo-db that overlaps with an MIC in at least one ITMI trio. Each point denotes one de novo mutation site. Points are colored by the autosome. ITMI, Inova Translational Medicine Institute.

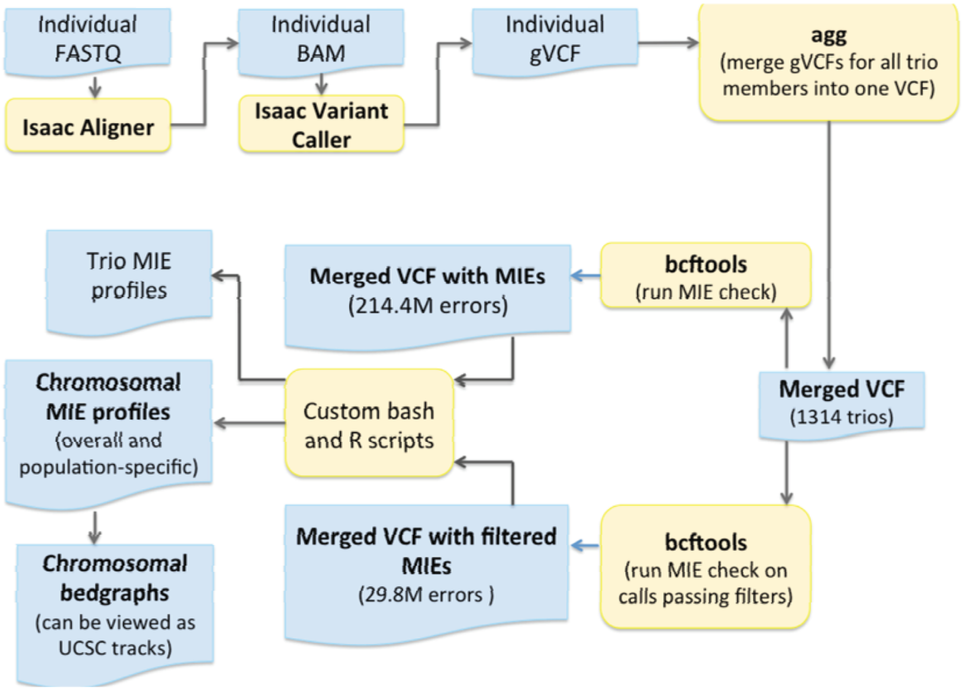

SUPPLEMENTARY FIG. S11. Analysis workflow.
